# Supplementary material for: Asian-white disparities in obstetric anal sphincter injury: Protocol for a systematic review and meta-analysis
Source: PLoS One. 2023 Sep 8;18(9):e0291174. doi: 10.1371/journal.pone.0291174 (PMC10490831; doi:10.1371/journal.pone.0291174)
Supplement: S2 File — Controlled vocabulary terms related to race, ethnicity and OASI. (DOCX) [file pone.0291174.s002.docx]

**S2 File. Systematic review search strategy**

| 1 | Asia* OR central asia* OR East* asia* OR South-East Asia* OR SouthEast Asia* OR South* Asia* OR West* Asia* OR Kazakh* OR Kyrgyz* OR Tajik* OR Turkmen* OR Uzbek* OR Chin* OR HongKong OR Maca* OR Korea* OR Mongolia* OR Japan* OR Brunei Darussalam* OR Cambodia* OR Indonesia* OR Lao* OR Malaysia* OR Myanmar* OR burm* OR Philippin* OR Singapor* OR Thai* OR Timor-Leste* OR Timorese* OR vietnam* OR Afghanistan* OR Bangladesh* OR Bhut* Sharchop* OR India* OR Iran* OR Maldive* Dhivehin* OR nepal* OR pakistan* OR sri lanka* OR west* asia* OR Armenia* OR Azerbaijan* OR Bahrain* OR Cypr* OR Georgia* OR Iraq* OR israel* OR jordan* OR kuwait* OR leban* OR oman* OR qatar* OR saudia arabia* OR turk* OR Emirate* OR yemen* |
| --- | --- |
| 2 | Obstetric trauma* OR Obstetric Anal Sphincter Injur* OR third-degree perineal laceration* OR third degree perineal laceration* OR third-degree tear* OR third degree tear* OR third-degree laceration* OR third degree laceration* OR obstetric anal sphincter laceration* OR obstetric anal sphincter tear* OR fourth-degree tear* OR fourth degree tear* OR fourth-degree perineal laceration* OR fourth degree perineal laceration* OR severe perineal laceration* OR perineal tear* OR obstetric injur* OR perineal injur* OR perineum injur* |
| 3 | 1 AND 2 |

Database: MEDLINE, OVID, Embase, Emcare and the Cochrane.
